# From the President’s Desk: Part 1, 2026

**DOI:** 10.4102/safp.v68i1.6320

**Published:** 2026-02-20

**Authors:** Tasleem Ras

**Affiliations:** 1 Division of Family Medicine, Department of Family, Community and Emergency Care, Faculty of Health Sciences, University of Cape Town, Cape Town, South Africa; 2 President and Chair of Executive Committee, South African Academy of Family Physicians, Durbanville, South Africa

As we move into the new year, we realise that some of the challenges of previous years have remained. These challenges align well with the strategic priorities defined at the strategic planning session of the South African Academy of Family Physicians (SAAFP) in December 2024. They were to: (1) expand the membership base; (2) strengthen alliances with other organised groupings in South Africa; (3) add value to the broader South African medical community; (4) work towards getting full recognition of specialist family physicians with private sector funders; and (5) enhance the financial stability of the SAAFP. There has been progress made in each of these domains, and we hope that we will see steady progression during 2026.

Our membership base has shown steady growth, and currently we have well over 400 members, with almost 300 being fully paid-up, and almost 100 trainee members. One of the areas we hope to see even more expansion in, is in associate memberships offered to general practitioners. The alliance with general practitioners and general practitioner-groups is going to become more important as we move towards developing a coherent picture of what primary care could look like under a single funder such as the proposed National Health Insurance (NHI).

We have taken strong steps in building sustainable relationships with other organisations and will be co-hosting a hugely exciting merged primary healthcare conference on 14–15 August 2026 in Midrand, Gauteng. Our partners include the Rural Doctors Association of South Africa, Rural Nursing South Africa, and Rural Rehab South Africa. We are hopeful that this collaboration will lead to substantive workstreams that will facilitate collective advocacy in strengthening primary healthcare across urban and rural settings.

Given the paucity of consultant family physician posts in the public sector, we need to sharpen our focus on the private sector. Specialist family physicians in private practice, as an organised collective within the SAAFP, have engaged the services of a professional consulting company to guide and facilitate negotiations with private medical aid administrators. This has led to a series of in-depth conversations with senior managers in the private sector, and we have now developed a much deeper understanding of the needs and perspective of the private funders. In addition, members are engaging with corporate private service providers in developing and implementing ‘hospital at home’ services. This is an exciting new space opening in community-based healthcare, and one which could very well become a niche area for family physicians. In 2026, we are hoping to initiate some regional meetings so that colleagues in private practice can get to know each other more personally.

I am pleased to report that the financial situation of the SAAFP has improved substantively, as presented by the Treasurer at our last Annual General Meeting (AGM). This is largely because of our enhanced revenue streams via the membership drive that has borne fruit, the acquisition of a grant that is funding some of our activities, and the SAAFP being positioned as an expert service provider for delivering faculty development to universities. Furthermore, we are involved in ongoing efforts to rationalise our expenditure patterns.

While the SAAFP will not be making any significant strategic changes over the course of the next year, we are currently well positioned to continue making an impact in multiple sectors across academia, public and private healthcare systems, and at the level of communities. I welcome all of you to join us as we continue this growth trajectory, ensuring that our voices help to shape the future of our health and healthcare systems.

